# Integrative analysis reveals gut microbiota-associated host regulatory networks in liver fibrosis

**DOI:** 10.3389/fcimb.2026.1963640

**Published:** 2026-10-13

**Authors:** Shiyu Peng, Yingying Nie, Yuzhu Song, Han Ding, Huiling Cao, Hao Wu, Jianni Qi, Qiang Zhu

**Affiliations:** 1 Department of Gastroenterology, The First Affiliated Hospital of Xinjiang Medical University, Urumqi, Xinjiang, China; 2 Department of Gastroenterology, The First Affiliated Hospital of Shihezi University, Shihezi, Xinjiang, China; 3 Department of Infectious Diseases, Shandong Provincial Hospital Affiliated to Shandong First Medical University, Jinan, Shandong, China; 4 Department of Central Laboratory, Shandong Provincial Hospital Affiliated to Shandong First Medical University, Jinan, Shandong, China

**Keywords:** gut microbiota, gut–liver axis, liver fibrosis, microbial metabolites, transcriptomics

## Abstract

**Background:**

Gut microbiota-derived metabolites may contribute to liver fibrosis by affecting host inflammatory, metabolic, and epigenetic pathways. We aimed to identify microbiota-associated host genes shared across liver fibrosis of different etiologies.

**Methods:**

Microbiota- and metabolite-associated host genes were obtained from gutMGene v2.0 and integrated with differentially expressed genes from two human liver transcriptomic datasets: GSE84044 for HBV-related fibrosis and GSE49541 for NAFLD-related fibrosis. Shared genes were analyzed using protein–protein interaction and functional enrichment analyses. Selected metabolite–protein interactions were assessed by molecular docking and molecular dynamics simulations. Two-sample Mendelian randomization was used to examine potential associations between candidate gene expression and liver fibrosis risk.

**Results:**

Fourteen microbiota-associated genes were differentially expressed in both fibrosis cohorts, including CCL2, CCL20, CXCL6, PTGS2, HDAC2, HDAC3, ADRA2A, SOD1, PEMT, and GNMT. These genes were mainly associated with chemokine activity, histone deacetylation, and IL-17 and TNF signaling. Molecular docking suggested structural compatibility between phenylacetylglutamine and ADRA2A, while butyrate showed weaker predicted interactions with HDAC2 and HDAC3.Molecular dynamics analysis supported the stability of the ADRA2A–phenylacetylglutamine complex under the simulated conditions. Mendelian randomization identified nominal associations of HDAC3, CCL2, PTGS2, and VCL expression with liver fibrosis risk.

**Conclusions:**

This study identified shared microbiota-associated host regulatory candidates in HBV- and NAFLD-related liver fibrosis.HDAC3 and ADRA2A may represent potential links between microbial metabolic signals and fibrotic responses, although further experimental validation is required.

## Introduction

Liver fibrosis is an aberrant wound-healing response to persistent liver injury and a common feature of chronic liver diseases, including chronic viral hepatitis, nonalcoholic fatty liver disease, alcohol-related liver disease, and autoimmune liver disease. It is characterized by hepatic stellate cell activation, myofibroblast accumulation, and excessive extracellular matrix deposition, which gradually disrupt liver architecture and impair hepatic function (Bataller and Brenner, 2005; Mederacke et al., 2013). Fibrosis can regress to some extent, as a subset of activated myofibroblasts may return to a relatively quiescent state once the underlying injury is removed (Kisseleva et al., 2012). If left uncontrolled, however, it may progress to cirrhosis, portal hypertension, and hepatocellular carcinoma. In nonalcoholic fatty liver disease, fibrosis stage is a major histological predictor of liver-related complications and long-term mortality (Angulo et al., 2015). Although antiviral therapy, weight reduction, and management of metabolic risk factors can slow disease progression in some patients, no treatment currently achieves reliable fibrosis reversal across different etiologies (Roehlen et al., 2020). Identifying molecular mechanisms shared by different forms of liver fibrosis may therefore help uncover therapeutic targets with broader clinical relevance.

The gut and liver communicate through continuous anatomical and functional interactions. Gut-derived nutrients, microbial components, and metabolites reach the liver through the portal circulation, while liver-derived bile acids shape the intestinal microenvironment, forming a dynamic bidirectional gut–liver communication system (Tripathi et al., 2018). Disruption of intestinal barrier integrity and gut microbial dysbiosis can increase hepatic exposure to microbe-associated molecular patterns (MAMPs), such as lipopolysaccharide. These microbial signals activate Toll-like receptor pathways and downstream inflammatory responses, promoting hepatic stellate cell activation and extracellular matrix deposition (Seki et al., 2007; Henao-Mejia et al., 2012). Clinical studies have identified distinct gut microbial alterations in patients with cirrhosis and advanced fibrosis associated with nonalcoholic fatty liver disease, suggesting that microbial composition is closely linked to fibrosis severity (Qin et al., 2014; Loomba et al., 2017). However, gut microbiome composition is influenced by numerous factors, including diet, medication use, age, comorbidities, and liver function, making it difficult to determine whether microbial changes contribute to fibrosis progression or represent secondary consequences of disease. Therefore, focusing on microbial metabolites and their host targets may provide a more mechanistic approach to understanding how gut microbial alterations influence liver fibrosis.

Gut microbiota-derived metabolites, including short-chain fatty acids and aromatic amino acid derivatives, participate in host metabolism and also function as signaling molecules that regulate immune responses and gene expression. Short-chain fatty acids modulate inflammatory processes and regulatory T-cell differentiation through G protein-coupled receptors, histone deacetylases, and immune cell metabolic pathways (Maslowski et al., 2009; Arpaia et al., 2013; Chang et al., 2014). In addition, gut-derived phenylacetylglutamine can influence host physiology through adrenergic receptor signaling (Nemet et al., 2020). These findings suggest that microbial metabolites can directly interact with host receptors, enzymes, and transcriptional regulators, providing a potential molecular link between intestinal microbial activity and responses in distant organs.

Liver fibrosis involves complex interactions among immune activation, inflammatory signaling, oxidative stress, and epigenetic regulation. Chemokine-mediated immune cell recruitment and cytokine pathways, including TNF and IL-17 signaling, contribute to the establishment of a profibrotic microenvironment. IL-17 signaling can activate Kupffer cells and hepatic stellate cells, thereby promoting inflammatory responses and collagen deposition (Meng et al., 2012; Tan et al., 2013). In addition, specific histone deacetylases have been implicated in hepatic stellate cell activation through regulation of chromatin states and fibrosis-related gene expression. Therefore microbial metabolites may influence liver fibrosis progression by simultaneously modulating inflammatory, chemotactic, and epigenetic pathways.

Most current studies investigating the relationship between gut microbiota and liver fibrosis have focused on individual disease entities or single transcriptomic datasets, making it difficult to distinguish shared regulatory mechanisms from disease-specific or cohort-specific effects. Hepatitis B virus (HBV)-related liver fibrosis is mainly driven by persistent viral infection and immune-mediated liver injury, whereas fibrosis associated with nonalcoholic fatty liver disease (NAFLD) is strongly linked to lipotoxicity, insulin resistance, and metabolic inflammation. Despite these distinct initiating factors, both conditions converge on common pathological processes, including chronic inflammation, hepatic stellate cell activation, and extracellular matrix deposition. The GSE84044 dataset provides transcriptomic profiles of HBV-related liver fibrosis (Wang et al., 2017), while GSE49541 contains liver transcriptomic data from NAFLD patients with different fibrosis stages (Murphy et al., 2013; Moylan et al., 2014). Identifying microbiota-associated host genes with consistent alterations across these independent cohorts may help minimize disease-specific bias and reveal conserved regulatory pathways involved in liver fibrosis progression.

Building on these observations, we integrated the gutMGene v2.0 database (Qi et al., 2025) with two independent human transcriptomic cohorts of HBV-related and NAFLD-related liver fibrosis to identify microbiota-associated host genes shared across different etiologies. Protein–protein interaction networks and functional enrichment analyses were subsequently performed to characterize the biological processes associated with these genes, particularly those related to immune regulation, inflammation, oxidative stress, and epigenetic control. Representative microbial metabolite–host protein interactions were further evaluated using molecular docking and 100-ns molecular dynamics simulations, while MR analyses were conducted to explore potential causal associations between candidate gene expression and liver fibrosis risk (Võsa et al., 2021). Similar integrative strategies combining transcriptomic analysis, microbial metabolite prediction, structural modeling, and genetic inference have recently been used to investigate microbe–host regulatory relationships (Li et al., 2026). In this study, we identified 14 core genes conserved across fibrosis etiologies. Our findings suggest that chemokine signaling, inflammatory pathways, epigenetic regulation, and oxidative stress may collectively contribute to the host response to gut microbiota-related metabolic signals during liver fibrosis progression.

## Materials and methods

### Study design

In this study, we applied an integrative bioinformatics approach to investigate the potential regulatory roles of gut microbiota-and metabolite-associated host targets in liver fibrosis. The overall workflow consisted of the following steps: (1) experimentally validated gut microbiota–host gene and microbial metabolite–host gene associations were obtained from the gutMGene v2.0 database; (2) differential expression analyses were performed using two independent human transcriptomic cohorts of HBV-related liver fibrosis and NAFLD-related liver fibrosis; (3) microbiota-associated candidate genes conserved across different etiologies were identified through three-way intersection analysis; (4) protein–protein interaction networks were constructed, followed by functional enrichment analyses; (5) molecular docking and molecular dynamics simulations were performed to evaluate the structural interactions between representative microbial metabolites and host target proteins; and (6) Mendelian randomization(MR) analyses based on cis-expression quantitative trait loci were conducted to assess potential causal associations between candidate gene expression and susceptibility to liver fibrosis (**Figure 1**).

**Figure 1:**
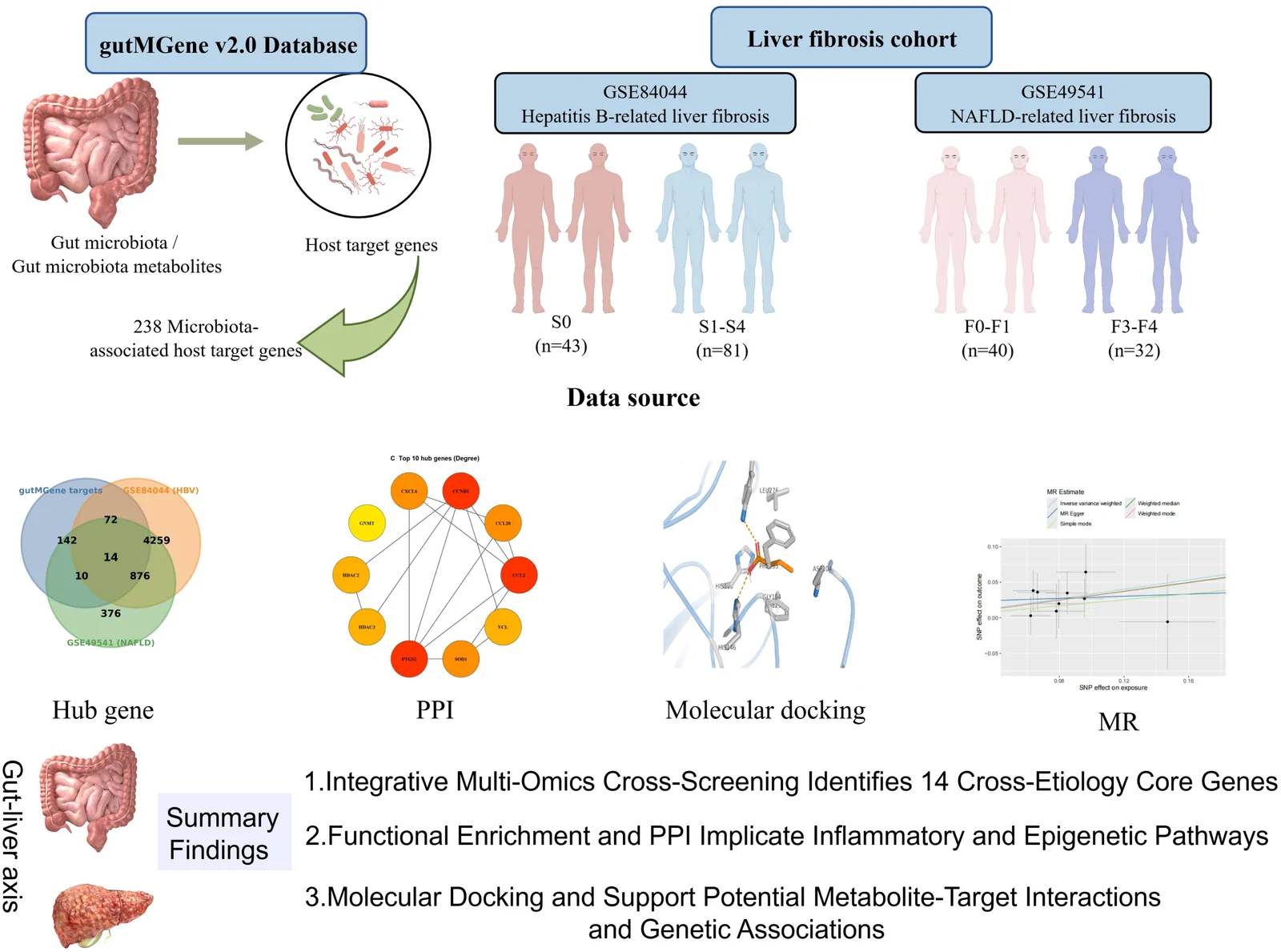
Workflow of the integrative multi-omics analysis in this study (by Figdraw).

### Identification of gut microbiota-related host gene

Associations among gut microbiota, microbial metabolites, and host genes were obtained from the gutMGene v2.0 database, which contains experimentally validated microbe–host gene and metabolite–host gene relationships (Qi et al., 2025). Only Homo sapiens records were included, and host target genes were extracted from the corresponding interaction tables. After removal of duplicate entries, non-human records, and associations without defined host targets,238 microbiota-associated host genes were retained for subsequent intersection analysis with liver fibrosis-related differentially expressed genes. For representative metabolites selected for structural analysis, chemical information, including canonical names, Canonical SMILES, and molecular structures, was obtained from the PubChem database.

### Acquisition and preprocessing of liver fibrosis transcriptomic datasets

Liver fibrosis-related transcriptomic datasets were obtained from the Gene Expression Omnibus (GEO) database of NCBI. Two independent human liver tissue cohorts with different etiologies were included:GSE84044 (HBV-related fibrosis, n=124) and GSE49541 (NAFLD-related fibrosis, n=72). Clinical characteristics of the included cohorts, including age, sex distribution, sample size, and fibrosis stage distribution, were summarized based on the original GEO annotations and are provided in **Supplementary Table S1**. In GSE84044, S0 samples were defined as the non-fibrosis group and S1–S4 samples as the fibrosis group according to Scheuer staging (Wang et al., 2017). In GSE49541, F0–F1 and F3–F4 samples were classified as mild and advanced fibrosis groups, respectively (Murphy et al., 2013; Moylan et al., 2014). Both datasets were generated using the Affymetrix Human Genome U133 Plus 2.0 Array platform. Expression matrices and annotation files were downloaded from GEO, and probe IDs were converted to official gene symbols. Probes without valid gene annotations were removed, and multiple probes mapping to the same gene were summarized by mean expression values. The two cohorts were analyzed independently without direct integration to minimize potential batch effects.

### Differential expression analysis

Differential expression analysis was performed using the limma package in R (Ritchie et al., 2015). Separate design matrices were constructed for GSE84044 and GSE49541, and differential expression was assessed using linear models with empirical Bayes moderation. Raw P values were adjusted using the Benjamini–Hochberg method. Genes with |log_2_FC| >0.585 and adjusted P<0.05 were considered significantly differentially expressed genes (DEGs). The two cohorts were analyzed independently, and the results were visualized using volcano plots, with upregulated, downregulated, and non-significant genes displayed separately.

### Protein–protein interaction network and hub gene identification

Three gene sets were intersected to identify microbiota-associated host genes shared across liver fibrosis etiologies:238 gut microbiota-related host genes from the gutMGene v2.0 database, DEGs from the HBV-related fibrosis cohort (GSE84044), and DEGs from the NAFLD-related fibrosis cohort (GSE49541). Genes overlapping among all three datasets were defined as “core genes” and were used for subsequent network, enrichment, structural, and genetic analyses.

The cross-etiology core genes were analyzed using the STRING database with Homo sapiens as the selected organism and a minimum interaction score of 0.4 (Szklarczyk et al., 2023). The resulting PPI network was visualized using the igraph package in R. Within the core gene PPI network, hub genes were defined as genes with the highest connectivity based on node degree ranking. The top 10 genes ranked by degree were selected as hub genes for network interpretation.

### Gene ontology and KEGG pathway enrichment analyses

Functional enrichment analyses were performed using the clusterProfiler package in R (Wu et al., 2021). Gene symbols were converted to Entrez Gene IDs before GO and KEGG analyses. GO analysis included biological process, cellular component, and molecular function categories, while KEGG analysis was performed using Homo sapiens as the reference organism. Enrichment significance was evaluated using the hypergeometric test with Benjamini–Hochberg correction, and terms with P < 0.05 and q < 0.20 were considered significant.

### Molecular docking analysis

Molecular docking was performed using AutoDock Vina 1.2.5 to assess potential interactions between representative microbial metabolites and host target proteins (O’Boyle et al., 2011; Eberhardt et al., 2021). The metabolite–protein pairs selected for docking were screened from gutMGene v2.0-derived metabolite–host gene associations according to biological relevance, availability of suitable human protein structures, and feasibility of small-molecule binding-site identification. Based on these criteria, PAGln–ADRA2A, butyrate–HDAC2/HDAC3, and diosgenin–SOD1 were selected for molecular docking analysis. Among these complexes, PAGln–ADRA2A and diosgenin–SOD1 showed relatively favorable docking scores and were further selected as representative complexes for molecular dynamics simulations. Protein structures were obtained from the RCSB Protein Data Bank, and ligand structures were retrieved from PubChem. The analyzed complexes included phenylacetylglutamine–ADRA2A (PDB ID: 6KUY), butyrate–HDAC2 (PDB ID: 4LXZ), butyrate–HDAC3 (PDB ID: 4A69), and diosgenin–SOD1 (PDB ID: 5YTO). SOD1 was selected because it was one of the core genes identified from the intersection of gutMGene-associated host genes and fibrosis-related transcriptomic datasets, and a corresponding diosgenin–SOD1 association was available in gutMGene. The SOD1 structure was obtained from the human wild-type SOD1 crystal structure (PDB ID: 5YTO, chain H), with the docking search region defined according to the original ligand binding geometry. Docking was performed with a grid size of 22Å×22Å×22Å, exhaustiveness=8, num_modes=9, and cpu=4. The lowest-energy poses were selected for visualization and interaction analysis using PyMOL.

### Molecular dynamics simulation

Molecular dynamics simulations were performed using GROMACS 2026 to evaluate the stability of representative protein–ligand complexes identified by molecular docking (Abraham et al., 2015). Protein and ligand parameters were generated using the AMBER99SB-ILDN and GAFF force fields, respectively, with AM1-BCC charges assigned to ligands. Systems were solvated with the TIP3P water model, neutralized with ions, and equilibrated through 100 ps NVT and 100 ps NPT simulations before a 100 ns production run with a 2 fs time step. Trajectory analyses included RMSD, RMSF, radius of gyration, protein–ligand distance, and hydrogen-bond interactions to evaluate the dynamic stability of the complexes.

### MR analysis

Two-sample MR analysis was performed using whole-blood cis-eQTL instruments from the eQTLGen Consortium (n = 31,684 individuals) to evaluate potential causal associations between core gene expression and liver fibrosis risk (Võsa et al., 2021). SNPs reaching genome-wide significance (P< 5×10^-8^) were selected and subjected to allele harmonization and LD clumping. Wald ratio or IVW methods were applied according to the number of available instruments, with weighted median and MR-Egger analyses as sensitivity tests (Hemani et al., 2018). Heterogeneity and horizontal pleiotropy were assessed using Cochran’s Q test, MR-Egger intercept, and MR-PRESSO (Verbanck et al., 2018). Effect estimates were reported with 95% confidence intervals, and P < 0.05 was considered significant.

### Statistical analysis

All transcriptomic and functional analyses were performed using R software (version 4.2.2). Differential expression analysis was conducted using the limma package, and GO/KEGG enrichment analyses were performed using the cluster Profiler package. Multiple testing correction was performed using the Benjamini–Hochberg method. DEGs were defined as |log_2_FC| > 0.585 and adjusted P < 0.05, while GO and KEGG enrichment significance was set at P<0.05 and q<0.20. MR analyses were performed using two-sided tests, with P<0.05 considered statistically significant. Figures were generated using R, PyMOL, and related trajectory analysis tools.

## Results

### Differentially expressed genes in two liver fibrosis cohorts

To identify transcriptional alterations shared across different etiologies of liver fibrosis, differential expression analyses were performed separately in the HBV-related fibrosis cohort (GSE84044) and the NAFLD-related fibrosis cohort (GSE49541). Principal component analysis and overall expression patterns indicated distinct transcriptomic differences between fibrosis groups in both cohorts.

Using the predefined criteria of |log_2_FC| >0.585 and adjusted P<0.05, 5221 DEGs were identified in GSE84044 and 2276 DEGs were identified in GSE49541. Volcano plots showed that both cohorts contained a substantial number of upregulated genes accompanied by a subset of downregulated genes, although the magnitude and distribution of expression changes differed between the two etiological backgrounds (**Figures 2A, B**).

**Figure 2:**
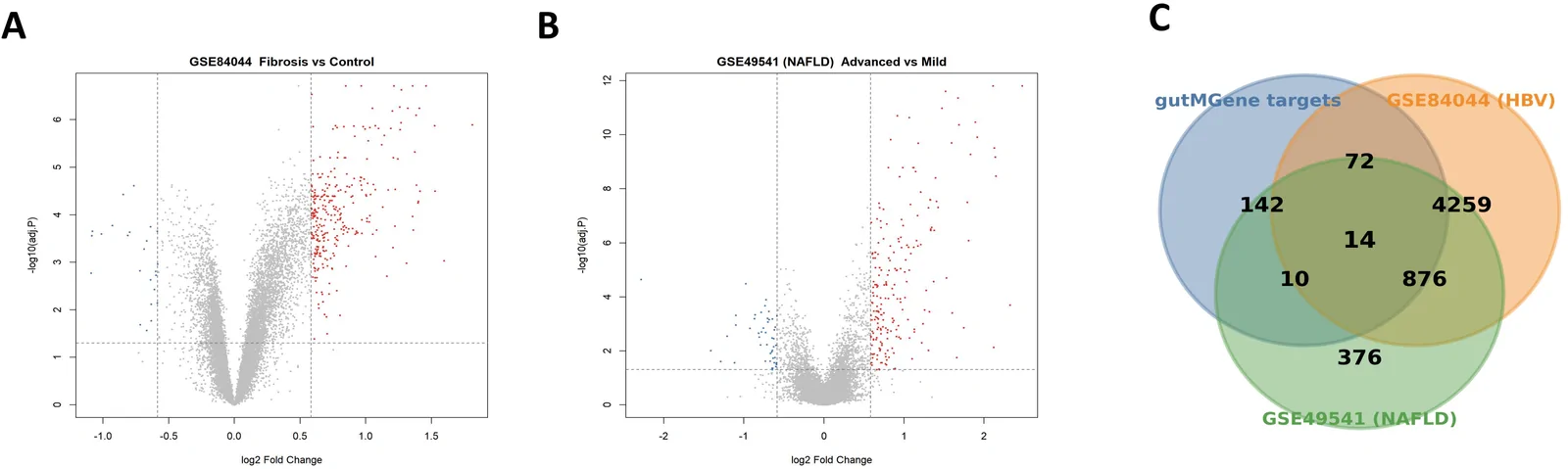
Differential expression analysis and identification of cross-etiology gut microbiota-associated core genes in liver fibrosis. **(A)** Volcano plot of DEGs between fibrotic and non-fibrotic groups in the HBV-related fibrosis cohort (GSE84044). **(B)** Volcano plot of DEGs between advanced and mild fibrosis groups in the NAFLD-related fibrosis cohort (GSE49541). Red and blue indicate upregulated and downregulated genes, respectively; gray indicates non-significant genes. **(C)** Venn diagram showing the overlap among gutMGene-derived microbiota-associated host genes and DEGs from GSE84044 and GSE49541. The 14 overlapping genes were defined as cross-etiology gut microbiota-associated core genes. FC, fold change; HBV, hepatitis B virus; NAFLD, non-alcoholic fatty liver disease.

Comparison of DEGs between the two cohorts identified 890 overlapping genes that were significantly altered in both HBV-related and NAFLD-related liver fibrosis. This shared gene set was subsequently integrated with gut microbiota-associated host genes for further candidate gene identification.

### Identification of cross-etiology gut microbiota-related core genes

A total of 238 experimentally documented host target genes associated with gut microbes or microbial metabolites were obtained from the gutMGene v2.0 database. (**Supplementary Table S2**)Intersection analysis of this gene set with DEGs from GSE84044 and GSE49541 identified 14 core genes that simultaneously showed microbiota-related evidence and differential expression in both HBV-related and NAFLD-related liver fibrosis cohorts (**Figure 2C**).

The 14 core genes included CXCL6, CCL20, CCL2, CCND1, ADRA2A, IGFBP6, VCL, PTGS2, BCL10, HDAC2, HDAC3, SOD1, PEMT, and GNMT. These genes were involved in diverse biological processes, including immune cell chemotaxis, inflammatory mediator production, cell cycle regulation, adrenergic signaling, epigenetic regulation, oxidative stress, and hepatic methyl donor metabolism.

Among the identified core genes, CXCL6 exhibited the largest expression change (log_2_FC=1.53, adjusted P = 1.3 × 10^-6^), followed by CCL20 (log_2_FC=1.08, adjusted P = 7.6×10^-4^) and CCL2 (log_2_FC=0.79, adjusted P = 8.4×10^-5^). CCND1, ADRA2A, IGFBP6, VCL, and PTGS2 also showed increased expression to varying degrees. In contrast, HDAC3, SOD1, PEMT, and GNMT displayed negative expression changes, with GNMT showing a relatively larger decrease (log_2_FC=−0.64, adjusted P = 2.3×10^-^³) (**Table 1**).

**Table 1:** Differential expression profiles of cross-etiology gut microbiota-associated core genes.

Gene	log_2_FC	Adjusted *p*
CXCL6	1.53	1.3e-06
CCL20	1.08	7.6e-04
CCL2	0.79	8.4e-05
CCND1	0.64	4.0e-05
ADRA2A	0.52	3.8e-05
IGFBP6	0.36	1.1e-03
VCL	0.30	1.4e-03
PTGS2	0.27	3.6e-02
BCL10	0.21	4.7e-03
HDAC2	0.13	1.9e-02
HDAC3	-0.12	1.5e-02
SOD1	-0.13	4.3e-04
PEMT	-0.17	9.1e-03
GNMT	-0.64	2.3e-03

### Protein interaction network and identification of hub genes

The 14 core genes were imported into the STRING database to construct a protein–protein interaction (PPI) network. The network showed functional connectivity among several core proteins, whereas nodes such as ADRA2A, BCL10, and IGFBP6 exhibited relatively fewer direct interactions with other proteins (**Figures 3A, B**).

**Figure 3:**
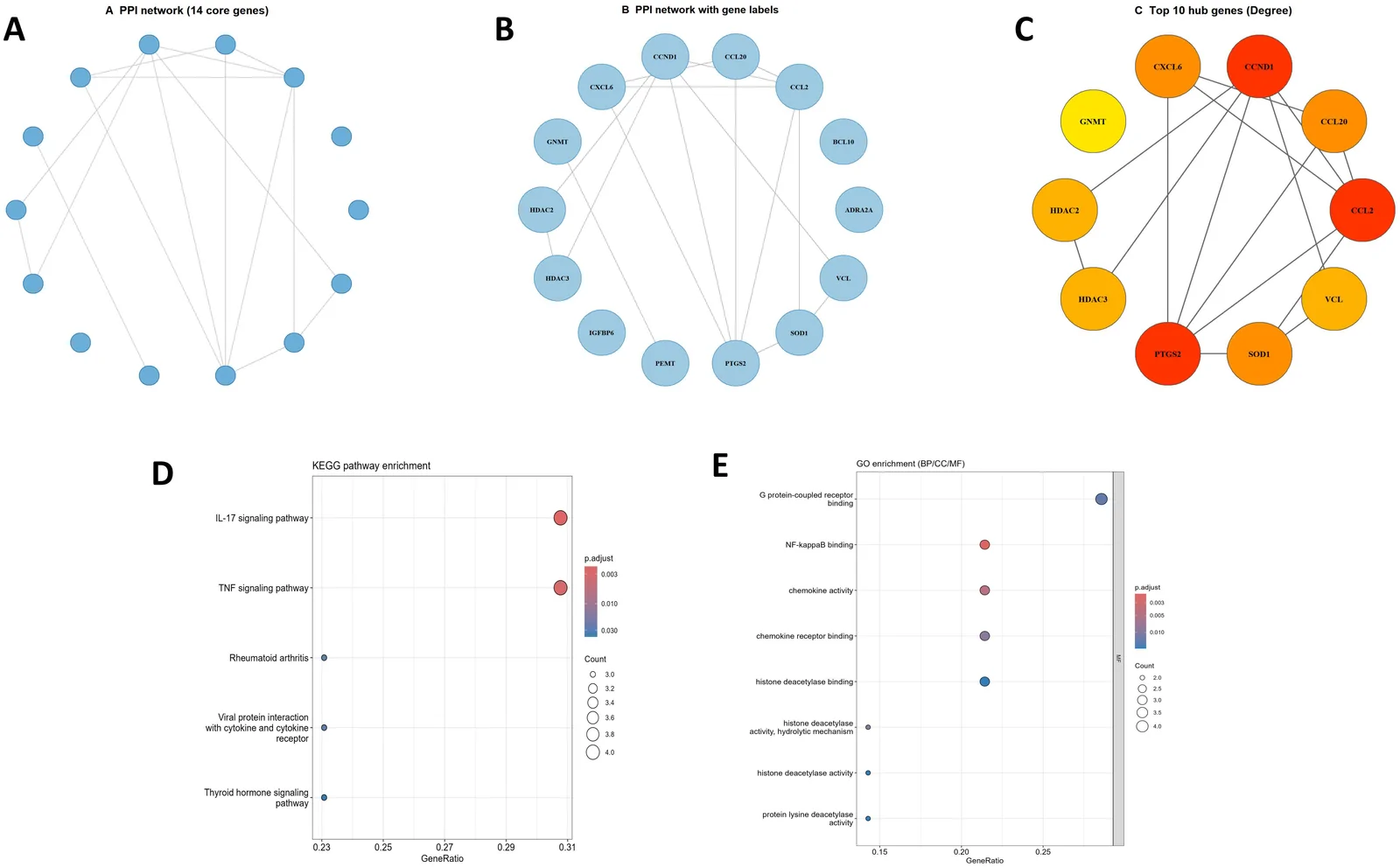
PPI network, hub genes, and functional enrichment analysis of cross-etiology core genes. PPI, protein–protein interaction; GO, Gene Ontology; KEGG, Kyoto Encyclopedia of Genes and Genomes. **(A)** PPI network constructed from the 14 core genes using STRING. Nodes represent proteins encoded by core genes, and edges indicate functional interactions. **(B)** PPI network with gene labels. **(C)** Top 10 hub genes ranked by node degree. Node color indicates increasing connectivity, with CCL2, CCND1, and PTGS2 showing the highest centrality. **(D)** KEGG enrichment analysis showing predominant enrichment in immune-related pathways, including IL-17 and TNF signaling pathways. **(E)** GO enrichment analysis showing enrichment in chemokine activity, chemokine receptor binding, NF-κB binding, G protein-coupled receptor binding, and histone deacetylase-related functions. Bubble size represents the number of core genes in each term, and color indicates adjusted P value.

Based on node degree ranking, CCL2, CCND1, and PTGS2 were identified as the top three hub genes with the highest connectivity (**Figure 3C**). CCL2 is mainly involved in chemokine-mediated immune cell recruitment, CCND1 participates in cell cycle regulation and proliferation, and PTGS2 links inflammatory mediator production with arachidonic acid metabolism. Other genes, including CXCL6, CCL20, VCL, SOD1, HDAC2, and HDAC3, were also located within major functional modules of the network. These network results suggest that gut microbiota-associated host genes are involved in multiple interconnected biological processes during liver fibrosis, including chemokine signaling, inflammatory responses, cell proliferation, oxidative stress, and epigenetic regulation, rather than being restricted to a single molecular pathway.

### Functional enrichment of the core genes

To further characterize the biological functions associated with the 14 core genes, GO and KEGG enrichment analyses were performed. GO molecular function analysis showed that the core genes were mainly enriched in terms related to chemokine activity, chemokine receptor binding, NF-κB binding, G protein-coupled receptor binding, histone deacetylase binding, histone deacetylase activity, and protein lysine deacetylase activity (**Figure 3D**). Among these terms, CCL2, CCL20, and CXCL6 mainly contributed to chemokine-related functions, whereas HDAC2 and HDAC3 were associated with histone deacetylation and protein lysine deacetylation processes. ADRA2A was primarily involved in G protein-coupled receptor binding and neurohumoral signaling regulation.

KEGG enrichment analysis indicated that the core genes were predominantly enriched in inflammation-and immune-related pathways, with IL-17 and TNF signaling pathways showing the strongest enrichment (**Figure 3E**). Genes includingCCL2, CCL20, CXCL6, and PTGS2 were involved in these pathways, suggesting that chemokine-mediated recruitment and inflammatory signal amplification may represent shared microbiota-associated host responses across different liver fibrosis etiologies. Although the epigenetic functions represented by HDAC2 and HDAC3 were not captured within a single KEGG pathway, they showed clear functional enrichment in GO analysis, suggesting that microbiota-associated metabolic signals may influence both inflammatory pathways and host chromatin regulation.

### Molecular docking between microbial metabolites and host targets

To evaluate the potential direct structural interactions between microbiota-associated metabolites and core host proteins, molecular docking was performed for three representative complexes: phenylacetylglutamine (PAGln)–ADRA2A, butyrate–HDAC2, and butyrate–HDAC3. PAGln showed the strongest predicted binding affinity with ADRA2A, with a binding energy of −8.0 kcal/mol, which was the lowest among the three docking systems (**Table 2**, **Supplementary Table S3**). The optimal docking pose indicated that PAGln occupied the orthosteric ligand-binding pocket of ADRA2A. Its polar groups formed hydrogen bonds with residues including ASP113, SER200, and SER204, while additional hydrophobic interactions were observed with surrounding pocket residues (**Figures 4A–C**). The ligand was accommodated within the transmembrane region without apparent steric clashes.

**Table 2:** Predicted binding energies of representative microbial metabolites and host target proteins.

Metabolite	Target protein	PDB ID	Binding energy (kcal/mol)
Phenylacetylglutamine (PAGln)	ADRA2A	6KUY	−8.0
Butyrate	HDAC3	4A69	−4.4
Butyrate	HDAC2	4LXZ	−4.5
Diosgenin	SOD1	5YTO	−7.39

PAGln, phenylacetylglutamine; ADRA2A, α2A-adrenergic receptor; HDAC2/3, histone deacetylase 2/3; SOD1, superoxide dismutase 1. Binding energies were predicted using AutoDock Vina. Lower binding energy values indicate stronger predicted binding affinity.

**Figure 4:**
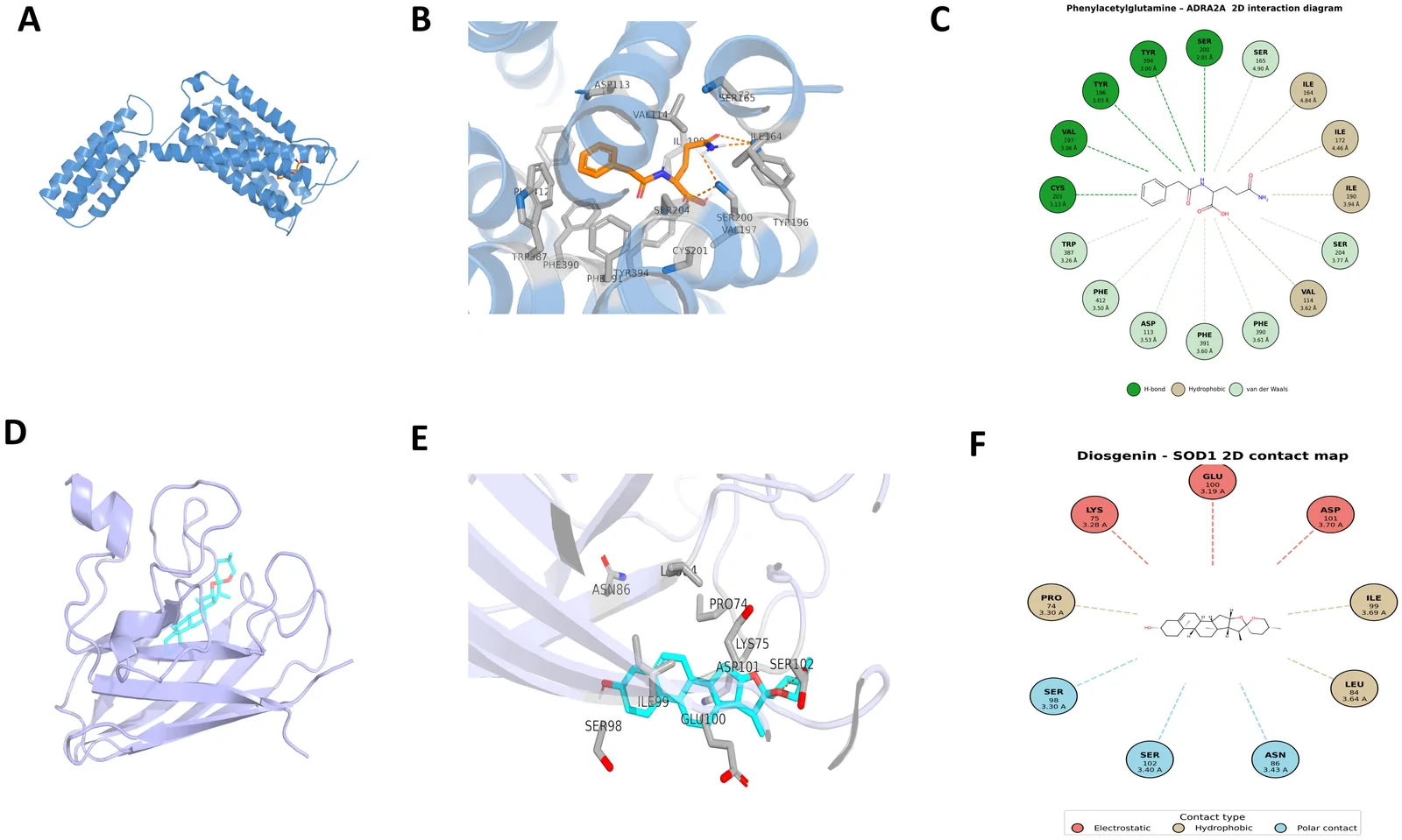
Molecular docking analysis of microbiota-associated metabolites and candidate host target proteins. PAGln, phenylacetylglutamine; ADRA2A, α2A-adrenergic receptor; HDAC2/3, histone deacetylase 2/3; SOD1, superoxide dismutase 1. Docking results indicate potential structural compatibility and do not represent experimentally confirmed direct binding. **(A)** Overall docking conformation of phenylacetylglutamine (PAGln) with ADRA2A. The protein is shown as a blue cartoon representation and the ligand as an orange stick model. **(B)** Three-dimensional interaction view of PAGln within the ADRA2A orthosteric binding pocket, showing hydrogen bonds and non-covalent interactions with key residues, including ASP113, SER200, and SER204. **(C)** Two-dimensional interaction diagram of the PAGln–ADRA2A complex, showing hydrogen bonds, hydrophobic interactions, and van der Waals contacts. The predicted binding energy was −8.0 kcal/mol. **(D)** Overall docking conformation of diosgenin with SOD1. The human wild-type SOD1 protein is shown as a blue cartoon representation, and diosgenin is shown as an orange stick model. The predicted binding energy was −7.39 kcal/mol. **(E)** Three-dimensional interaction view of diosgenin within the predicted SOD1 binding pocket, showing the spatial arrangement of the ligand and surrounding residues, including GLU100, LYS75, PRO74, SER98, SER102, ASN86, LEU84, ILE99, and ASP101. **(F)** Two-dimensional interaction diagram of the diosgenin–SOD1 complex, showing predicted hydrogen bonds, hydrophobic interactions, and other non-covalent contacts between diosgenin and SOD1 residues. Dashed lines indicate predicted interactions.

The predicted binding energies of butyrate with HDAC3 and HDAC2 were −4.4 and −4.5 kcal/mol, respectively. Docking poses showed that butyrate was positioned within the catalytic channels of both HDAC2 and HDAC3, near the catalytic zinc ion sites (**Figures 4D–F**). The carboxyl group of butyrate formed interactions with polar residues within the catalytic pocket. However, due to its smaller molecular size, butyrate exhibited fewer contacts and weaker predicted binding affinity compared with the PAGln–ADRA2A complex.

The PAGln–ADRA2A complex showed a more favorable predicted binding energy and a relatively complete interaction network within the binding pocket, whereas the butyrate–HDAC2/HDAC3 complexes demonstrated structural feasibility for occupying the catalytic channels but with relatively weaker predicted binding affinities. These docking results primarily support the structural plausibility of the proposed metabolite–protein interactions and do not independently demonstrate experimentally validated binding affinity or functional effects.

### Molecular dynamics simulations support stable retention of PAGln in the ADRA2A binding pocket

To further evaluate the dynamic stability of protein–ligand complexes, 100 ns all-atom molecular dynamics (MD) simulations were performed for the ADRA2A–PAGln complex and an additional candidate system, SOD1–diosgenin.

#### ADRA2A–PAGln complex

During the 100 ns simulation, the ADRA2A backbone underwent initial conformational adjustment and gradually reached a stable state, with an average backbone RMSD of approximately 0.62 nm. The RMSD of PAGln showed slight fluctuations during the early stage and stabilized at approximately 0.43 nm in the later phase without continuous increase.

The average protein–ligand minimum distance was approximately 0.19 nm, with a maximum distance of approximately 0.24 nm, indicating that PAGln remained close to the binding pocket throughout the simulation. Hydrogen-bond analysis showed that protein–ligand hydrogen bonds were maintained in approximately 95% of trajectory frames, with an average of two hydrogen bonds (**Figure 5A**). No persistent increase in RMSF was observed for key binding-site residues, and the radius of gyration remained relatively stable.

**Figure 5:**
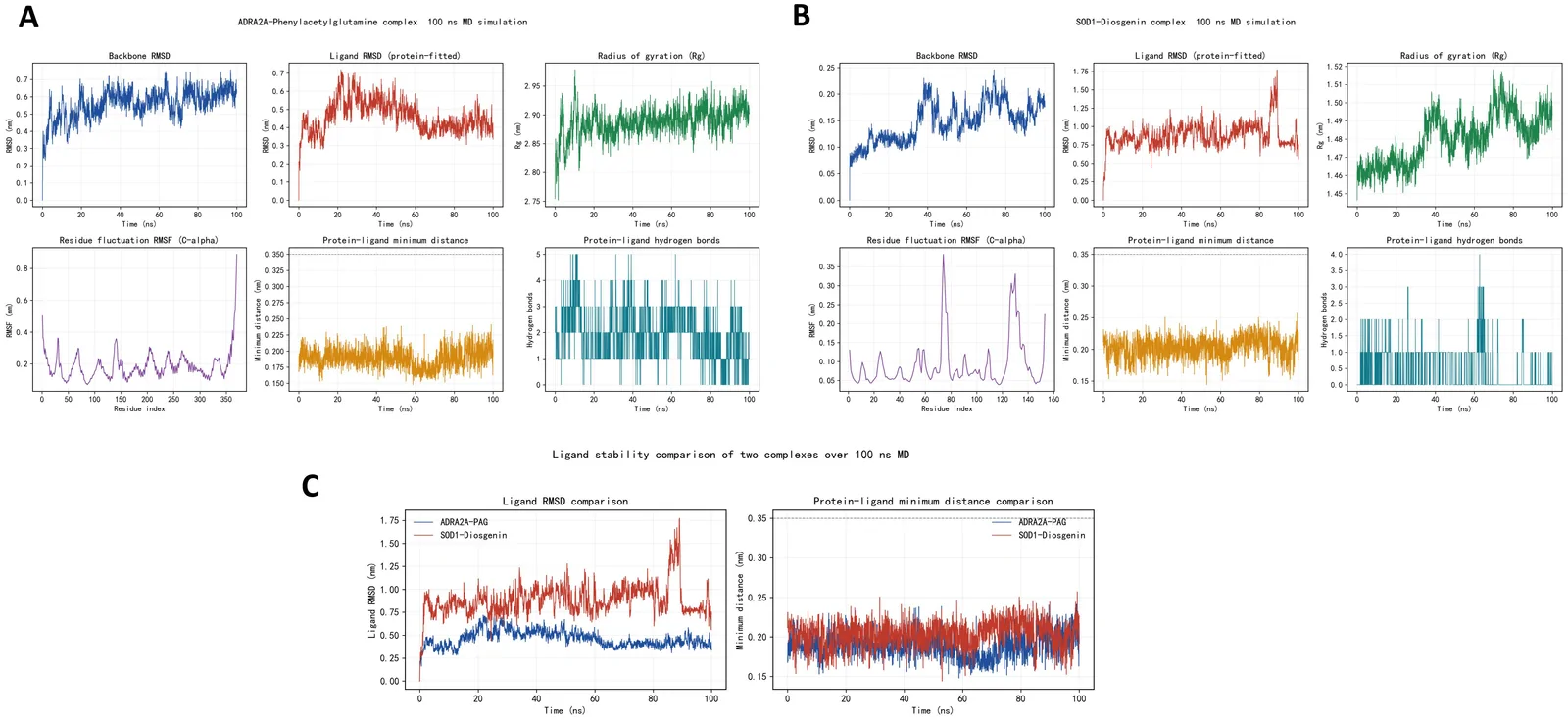
100 ns molecular dynamics simulations of ADRA2A–PAGln and SOD1–diosgenin complexes. RMSD, root mean square deviation; RMSF, root mean square fluctuation; Rg, radius of gyration; MD, molecular dynamics; SOD1, superoxide dismutase 1; PAGln, phenylacetylglutamine; Rg, radius of gyration. **(A)** MD trajectory analysis of the ADRA2A–PAGln complex, including protein backbone RMSD, ligand RMSD after protein fitting, radius of gyration (Rg), residue RMSF, protein–ligand minimum distance, and hydrogen-bond number. **(B)** Corresponding MD parameters of the SOD1–diosgenin complex. **(C)** Comparison of ligand RMSD and protein–ligand minimum distance between the two complexes. The ADRA2A–PAGln complex showed lower ligand RMSD and more stable protein–ligand distance, whereas the SOD1–diosgenin complex exhibited greater ligand fluctuations, suggesting conformational adjustment or binding-site migration during simulation.

These results suggest that PAGln maintained a stable interaction pattern within the ADRA2A orthosteric pocket after initial conformational adaptation, supported by persistent hydrogen-bond and hydrophobic interactions.

#### SOD1–diosgenin complex

In the SOD1–diosgenin system, the protein backbone RMSD remained approximately 0.18 nm, indicating overall structural stability of SOD1 during simulation. However, the ligand RMSD increased to approximately 0.92 nm during the later stage, which was substantially higher than that of the ADRA2A–PAGln system, suggesting considerable conformational adjustment or positional migration of diosgenin within the binding region (**Figure 5B**).

Despite ligand displacement, the average protein–ligand minimum distance remained approximately 0.20 nm. Hydrogen bonds were detected in approximately 37% of trajectory frames, lower than that observed for the ADRA2A–PAGln complex (**Figure 5C**). Trajectory analysis showed that diosgenin maintained contact with the SOD1 surface after moving away from the initial docking position, suggesting that hydrophobic interactions may contribute more than persistent hydrogen-bond networks to complex stabilization.

Overall, the SOD1–diosgenin complex exhibited stable protein conformation but ligand relocation during simulation, representing a shifted binding mode with weaker dynamic stability compared with the ADRA2A–PAGln complex (**Table 3**).

**Table 3:** Key molecular dynamics parameters of protein–ligand complexes during 100 ns simulations.

Complex	Protein RMSD (nm)	Ligand RMSD (late stage)	Minimum distance (nm)	H-bond occupancy	Interpretation
ADRA2A–PAGln	0.62	0.43	0.19	95%	Maintained within the binding pocket
SOD1–diosgenin	0.18	0.92	0.20	37%	Ligand displacement with hydrophobic contact maintenance

### MR analysis of core gene expression and liver fibrosis

Of the 14 cross-etiology core genes, 13 had eligible cis-eQTL instruments after SNP selection and linkage disequilibrium clumping and were included in two-sample MR analysis using liver fibrosis GWAS GCST90473873 as the outcome; CCL20 was excluded because no eligible instrument was available.

Inverse-variance weighted analysis showed that genetically predicted HDAC3 expression was positively associated with liver fibrosis risk (OR = 1.40, 95% CI: 1.08–1.80, P = 0.011). In contrast, higher genetically predicted expression of CCL2 (OR = 0.56, 95% CI: 0.37–0.85, P = 0.007), PTGS2 (OR = 0.92, 95% CI: 0.86–0.99, P = 0.025), and VCL (OR = 0.95, 95% CI: 0.91–1.00, P = 0.045) was associated with lower liver fibrosis risk. GNMT showed a suggestive inverse association (OR = 0.95, 95% CI: 0.90–1.00, P = 0.060). No significant associations were observed for CCND1, PEMT, HDAC2, SOD1, BCL10, IGFBP6, CXCL6, or ADRA2A (**Figure 6**; **Supplementary Table S4**).

**Figure 6:**
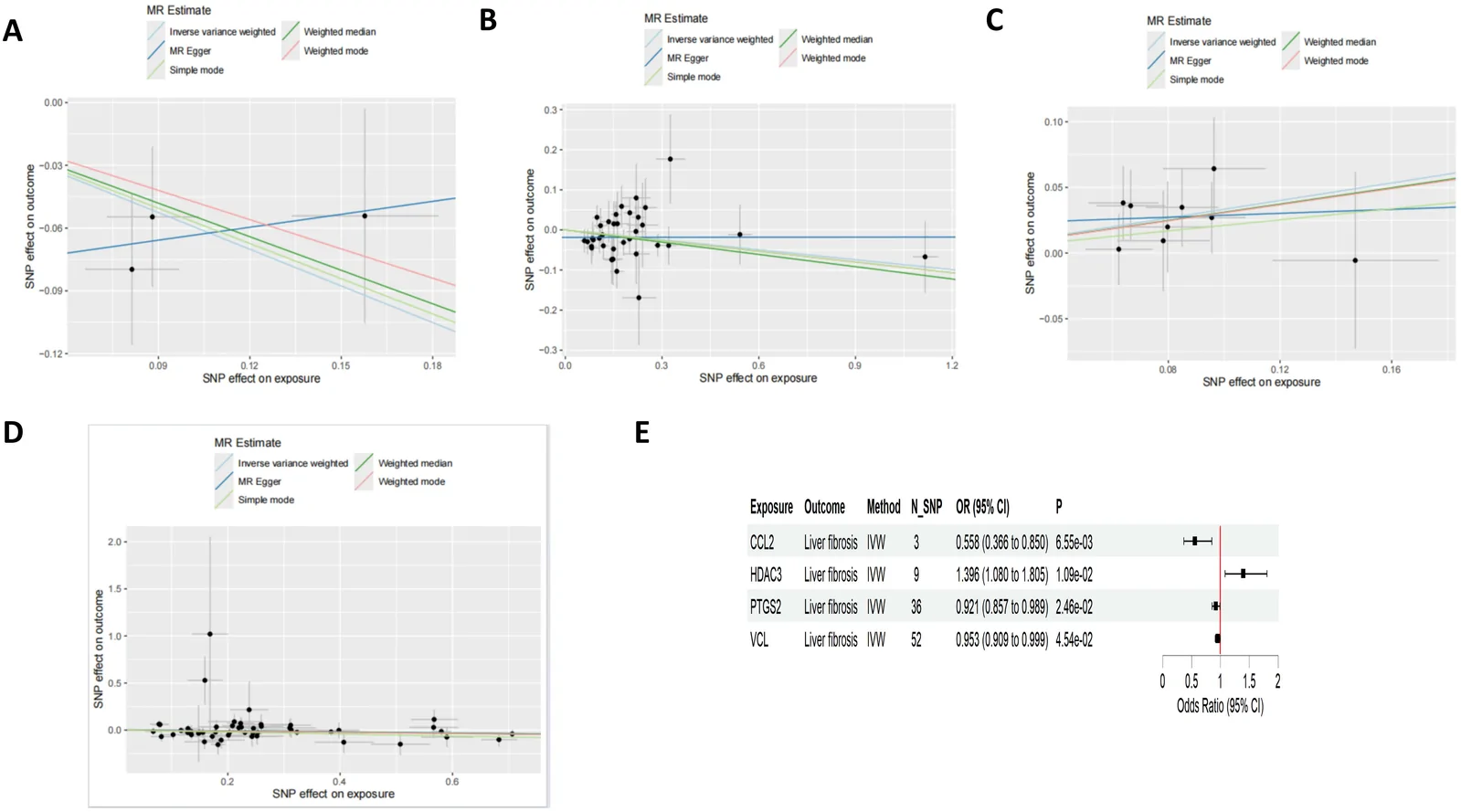
Mendelian randomization analysis of the associations between genetically predicted CCL2, HDAC3, PTGS2, and VCL expression and liver fibrosis risk. SNP, single nucleotide polymorphism; MR, Mendelian randomization; IVW, inverse-variance weighted; CCL2, C-C motif chemokine ligand 2; PTGS2, prostaglandin-endoperoxide synthase 2; HDAC3, histone deacetylase 3; VCL, vinculin. **(A–D)** MR scatter plots showing the associations of genetically predicted CCL2, PTGS2, HDAC3 and VCL expression with liver fibrosis outcomes. **(E)** Forest plot of Mendelian randomization. Each dot represents a genetic instrumental variable. The x-axis indicates the SNP effect on gene expression, and the y-axis indicates the SNP effect on liver fibrosis risk. Error bars represent uncertainty of effect estimates. Regression lines represent causal estimates derived from IVW, MR-Egger, weighted median, simple mode, and weighted mode methods.

These findings identify HDAC3, CCL2, PTGS2, and VCL as genes with nominal genetic associations with liver fibrosis susceptibility. Given the number of genes tested, the estimates should be interpreted as suggestive rather than definitive causal evidence.

## Discussion

In this study, we integrated gut microbiota-associated host targets, two independent human liver fibrosis transcriptomic cohorts, protein–protein interaction networks, functional enrichment analyses, molecular docking, molecular dynamics simulations, and MR analyses to establish a multi-layered evidence framework for investigating microbiota-associated metabolic signals in liver fibrosis. Among 238 microbiota-associated host genes,14 core genes were identified that were consistently altered in both HBV-related and NAFLD-related liver fibrosis cohorts. CCL2, CCND1, and PTGS2 showed high connectivity within the PPI network, while the core genes were mainly enriched in chemokine activity, histone deacetylation, G protein-coupled receptor binding, and IL-17/TNF signaling pathways. Structural analyses further suggested potential interactions between phenylacetylglutamine and ADRA2A, as well as the potential accommodation of butyrate within the catalytic pockets of HDAC2 and HDAC3. MR analyses provided genetic evidence supporting potential associations involving HDAC3, CCL2, and PTGS2. Collectively, these findings support a microbiota–host regulatory network involving immune chemotaxis, epigenetic regulation, neurohumoral signaling, and hepatic metabolic processes, rather than a simple linear axis driven by a single microbial species, metabolite, or target.

An important feature of this study is the identification of shared molecular signals across two liver fibrosis cohorts with distinct etiological backgrounds. HBV-related fibrosis is primarily driven by persistent viral infection and immune-mediated injury, whereas NAFLD-related fibrosis is more closely associated with lipotoxicity, insulin resistance, and metabolic inflammation. Despite these different initiating factors, both conditions ultimately converge on sustained hepatic inflammation, hepatic stellate cell activation, and extracellular matrix deposition (Bataller and Brenner, 2005; Murphy et al., 2013; Moylan et al., 2014; Wang et al., 2017; Roehlen et al., 2020). Therefore, microbiota-associated DEGs consistently identified in both cohorts may represent relatively stable host responses involved in liver fibrosis progression. This cross-etiology design reduces the influence of cohort-specific associations and shifts the focus from disease-specific markers toward potential regulatory nodes shared across chronic liver diseases. However, the fibrosis classifications differed between the two datasets. GSE84044 compared non-fibrotic samples with samples across different fibrosis stages, whereas GSE49541 compared mild and advanced fibrosis groups. Therefore, the 14 overlapping genes may be involved in either fibrosis initiation or progression, and the current cross-sectional datasets cannot clearly distinguish between these possibilities.

CCND1 was identified as another highly connected node in the PPI network, suggesting that cell proliferation-related processes may also contribute to fibrosis-associated responses. CCND1 encodes cyclin D1, a key regulator of the G1/S transition, and its abnormal regulation has been linked to proliferative responses and tissue remodeling during chronic injury. In the fibrotic liver, sustained inflammatory stimulation and metabolic disturbances can promote the activation and expansion of multiple hepatic cell populations, including hepatic stellate cells and infiltrating immune cells, thereby facilitating extracellular matrix deposition. Although direct regulation of CCND1 by microbial metabolites was not assessed in this study, gut-derived microbial signals may influence CCND1-related pathways indirectly through inflammatory responses, oxidative stress, and metabolic alterations. Further experimental studies are required to clarify whether CCND1 serves as a functional mediator connecting microbial signals and hepatic fibrogenesis.

The chemokine network represented one of the most prominent functional modules identified in this study. CCL2, CCL20, and CXCL6 were among the 14 core genes, and CCL2 also exhibited one of the highest connectivity values in the PPI network. The CCL2-CCR2 axis promotes the recruitment of circulating monocytes to injured liver tissue and facilitates the accumulation of inflammatory macrophages. In experimental steatohepatitis models, inhibition of this pathway has been shown to reduce hepatic macrophage infiltration and liver injury (Miura et al., 2012). CCL20 is also elevated in NAFLD fibrotic tissues and can be produced by hepatic stellate cells under fatty acid stress conditions (Chu et al., 2018). These previous findings are consistent with the chemokine-related enrichment patterns and increased expression of CCL2 and CCL20 observed in our study, suggesting that sustained immune cell recruitment may represent an important process linking gut microbiota-associated signals to hepatic fibrotic responses.

However, the role of chemokines in liver fibrosis is not always unidirectional. The CCL20 receptor CCR6 participates not only in inflammatory cell recruitment but also in the trafficking of specific γδ T cell subsets into injured liver tissue. In some chronic liver injury models, these cells can promote apoptosis of activated hepatic stellate cells and limit excessive fibrosis (Hammerich et al., 2014). Therefore, increased CCL20 expression may reflect both pro-inflammatory immune cell recruitment and the activation of compensatory immunoregulatory responses. The ultimate biological outcome likely depends on the cellular source of CCL20, the composition of recruited immune populations, disease stage, and the local cytokine environment. This context-dependent regulation also explains why tissue-level transcriptomic data alone cannot determine the net pro-fibrotic or anti-fibrotic effects of individual chemokines. Future studies integrating single-cell transcriptomics, spatial transcriptomics, and ligand–receptor interaction analyses will be required to define the cellular origins and target populations of CCL20, CCL2, and CXCL6 in hepatocytes, hepatic stellate cells, macrophages, and distinct lymphocyte subsets.

The enrichment of IL-17 and TNF signaling pathways further indicates that the core genes represent more than isolated chemokine responses and are interconnected with broader inflammatory amplification networks. Previous studies have shown that IL-17 can directly act on hepatic stellate cells and promote the release of inflammatory mediators and pro-fibrotic factors through Kupffer cells (Meng et al., 2012; Tan et al., 2013). TNF signaling is involved in immune cell activation, cell survival, and inflammatory gene transcription. The presence of CCL2, CCL20, CXCL6, and PTGS2 within these pathways suggests that gut-derived signals may initially influence hepatic immune cell composition and subsequently amplify hepatic stellate cell responses through cytokine and lipid mediator networks. Notably, enrichment analysis reflects the statistical overrepresentation of gene sets in known pathways and does not directly demonstrate pathway activation. Further experimental studies are required to assess the phosphorylation status of key signaling proteins, such as NF-κB, STAT3, and JNK, and to evaluate fibrosis-related endpoints including collagen deposition, α-SMA expression, and TIMP1 levels.

Beyond immune chemotaxis, the identification of HDAC2 and HDAC3 suggests that epigenetic regulation may represent another important layer through which microbiota-derived metabolites influence liver fibrosis. The transition of hepatic stellate cells from a quiescent state to a myofibroblast-like phenotype requires extensive and relatively stable transcriptional reprogramming. Previous studies have shown that HDAC3 promotes hepatic stellate cell activation by deacetylating targets and suppressing PPAR-γ signaling, whereas inhibition of HDAC3 can partially restore PPAR-γ activity and attenuate experimental liver fibrosis (Zhang et al., 2023). In the present study, HDAC3 received support from both cross-etiology transcriptomic analysis and MR evidence, making it one of the candidate genes with relatively convergent evidence. Recent studies have further demonstrated that hepatic stellate cell activation involves multiple metabolism-sensitive epigenetic modifications, including histone acetylation and lactylation, highlighting the close connection between cellular metabolic states and chromatin regulation (Rho et al., 2023).

Butyrate is a representative gut microbial metabolite with HDAC inhibitory activity and has been reported to regulate macrophage function and immune cell differentiation (Maslowski et al., 2009; Arpaia et al., 2013; Chang et al., 2014). In this study, molecular docking showed that butyrate could enter the catalytic channels of HDAC2 and HDAC3 and approach the catalytic zinc-containing regions, providing structural support for the potential model of a “microbial short-chain fatty acid–HDAC–host transcriptional regulation” axis. However, the predicted binding energies of butyrate with HDAC2 and HDAC3 were approximately−4.5 and −4.4kcal/mol, respectively, which were weaker than those typically observed in complexes with stronger predicted binding potential. Therefore, these results should be interpreted as evidence for structural accessibility and weak interaction potential rather than high-affinity or selective binding between butyrate and HDAC2/HDAC3. Due to its small molecular size, the biological effects of short-chain fatty acids are also influenced by local concentration, ionization state, cellular uptake, and competition among different HDAC isoforms. Further studies using purified enzyme activity assays, thermal shift assays, or isothermal titration calorimetry are required to validate direct binding and to compare the inhibitory effects of butyrate on HDAC2, HDAC3, and other HDAC isoforms.

The ADRA2A–phenylacetylglutamine (PAGln) interaction represents another noteworthy candidate relationship identified by structural analysis. Previous studies have demonstrated that PAGln, a gut microbiota-derived metabolite, can influence host physiological responses through adrenergic receptors (Nemet et al., 2020). In this study, PAGln showed a predicted binding energy of −8.0 kcal/mol with ADRA2A and remained close to the receptor binding pocket during molecular dynamics simulation, suggesting favorable structural compatibility between the metabolite and receptor. These findings extend the known relationship between microbiota-derived metabolites and adrenergic signaling toward a potential mechanism relevant to liver fibrosis. However, it remains unclear whether PAGln acts as an agonist, antagonist, or allosteric modulator of ADRA2A, and whether this interaction occurs in specific hepatic cell populations. The pocket stability observed during molecular dynamics simulations only indicates that the complex remained structurally stable within the defined force field and simulation timescale, rather than demonstrating intracellular receptor activation. Therefore, receptor-binding assays, second messenger analyses, and ADRA2A knockdown or antagonist experiments are required to determine the pharmacological properties of this interaction and its effects on hepatic stellate cells, hepatocytes, and intrahepatic immune cells.

MR analysis provided an evidence layer distinct from disease-associated tissue expression patterns. The eQTL instruments used here were derived from whole blood in the eQTLGen Consortium (Võsa et al., 2021). Although cis-eQTLs can be shared across tissues, their effect magnitudes and detectability may differ because of tissue- and cell-type-specific regulatory architecture. Accordingly, the MR estimates should be interpreted as effects of genetically proxied expression captured in blood rather than as direct estimates of liver-specific gene expression. Blood-based instruments may therefore underrepresent or miss regulatory effects that are specific to hepatocytes, hepatic stellate cells, or intrahepatic immune cells, despite the greater statistical power and instrument availability afforded by the large eQTLGen sample. Genetically predicted higher HDAC3 expression was directionally associated with increased liver fibrosis risk, which was consistent with previous experimental evidence indicating a role of HDAC3 in promoting hepatic stellate cell activation. In contrast, although CCL2 and PTGS2 showed increased expression in fibrosis tissues, MR analyses suggested an inverse association between genetically predicted expression levels and liver fibrosis risk. Such directional discrepancies do not necessarily indicate conflicting results. Increased expression in diseased tissues may represent a reactive response following tissue injury, whereas MR estimates reflect long-term genetically determined differences in gene expression. Therefore, these approaches capture different biological dimensions involving distinct temporal scales, tissue contexts, and interpretations.

The findings for PTGS2 particularly illustrate the dual roles of inflammatory genes in liver fibrosis. PTGS2 encodes cyclooxygenase-2 (COX-2), a rate-limiting enzyme involved in prostaglandin synthesis under inflammatory conditions; however, its downstream products do not uniformly exert pro-fibrotic effects. Previous *in vitro* studies have shown that prostaglandin E_2_ derived from COX-2 can inhibit TGF-β1-induced type I collagen expression in hepatic stellate cells (Hui et al., 2004). Therefore, increased PTGS2 expression in injured liver tissue may reflect both inflammatory activation and a compensatory response that limits excessive collagen production. This provides a potential biological explanation for the partial discrepancy between tissue-level PTGS2 expression and MR-derived genetic associations in this study. Similar context- and cell type-dependent effects may also apply to CCL2. However, its MR results may be influenced by differences between peripheral blood eQTL and liver tissue expression, limited numbers of instrumental variables, linkage disequilibrium, and horizontal pleiotropy. Definitive causal interpretation will require liver tissue-specific eQTL analyses, colocalization analyses, and multi-tissue MR approaches to determine whether the exposure and outcome associations are driven by the same causal variants.

Genes such as GNMT, PEMT, and SOD1 suggest that microbiota-associated host responses may also involve hepatic metabolic homeostasis and redox regulation. GNMT and PEMT are closely involved in hepatic methyl donor metabolism and phospholipid metabolism, and their reduced expression may reflect impaired one-carbon metabolism and membrane lipid metabolic capacity during chronic liver injury. Changes in SOD1 expression may be associated with alterations in reactive oxygen species scavenging capacity and oxidative stress burden. Compared with chemokine-related genes and HDAC3, these genes currently receive support mainly from transcriptomic evidence and lack an integrated metabolite–target–genetic evidence chain. Therefore, they should be considered candidate nodes for future metabolic investigations rather than definitive causal targets at the current stage.

This study has several methodological strengths. First, both transcriptomic cohorts were derived from human liver tissues and represented two major chronic liver disease contexts, namely viral and metabolic liver fibrosis. Each cohort was analyzed independently to avoid potential batch effects caused by directly integrating samples from different etiologies. Second, candidate genes were required to show significant alterations in both cohorts and to have microbiota or metabolite-associated evidence from the gutMGene database, thereby improving the relevance of the identified targets to the gut–liver axis. Third, transcriptomic analysis, network topology, structural modeling, and genetic inference provided complementary evidence from multiple levels, reducing the limitations associated with reliance on a single data source. Compared with approaches based solely on differential expression and enrichment analyses, this integrative evidence framework may help distinguish general inflammatory markers from candidate targets with greater potential for experimental validation. Previous studies have also combined transcriptomics, metabolite–target analyses, molecular simulation, and MR approaches; however, the present study highlights a more distributed multi-node regulatory network rather than a single dominant molecular axis.

### Limitations and future directions

Several limitations should be considered when interpreting the present findings. First, both transcriptomic datasets were cross-sectional microarray studies, limiting the ability to determine whether microbiota-associated gene alterations preceded or followed fibrosis development. In addition, individual-level covariates, including diet, medication, age, and metabolic status, were unavailable. Second, bulk liver transcriptomic profiles represent mixed signals from hepatocytes, hepatic stellate cells, endothelial cells, and various immune populations; therefore, increased expression of a given gene may reflect changes in cellular composition rather than enhanced transcription within individual cells. Third, gutMGene is based on previously reported experimental associations, and its coverage is influenced by current research availability and experimental conditions, potentially leading to underrepresentation of less-studied microbes and metabolites. Fourth, network centrality in PPI analysis is affected by existing literature abundance, and the high connectivity of CCL2, CCND1, and PTGS2 should not be directly interpreted as evidence of causal importance in liver fibrosis. Fifth, molecular docking and MD simulations depend on structural models, protonation states, binding-site definitions, and force-field parameters, and therefore only support computational plausibility of molecular interactions. In particular, the catalytic pockets of HDAC proteins contain metal ions, and conventional docking approaches may incompletely capture metal coordination effects. Sixth, MR analyses were based on whole-blood eQTLs from eQTLGen. Although many cis-eQTLs are shared across tissues, tissue-specific effect sizes and regulatory variants may differ; therefore, blood-derived instruments may not fully capture gene regulation in hepatocytes, hepatic stellate cells, or intrahepatic immune populations and may attenuate or miss associations driven by liver-specific regulation. Future studies should repeat the analyses using liver-specific cis-eQTLs where sufficiently strong instruments are available and incorporate colocalization or multi-tissue analyses. Seventh, this study lacked paired microbiome, circulating or fecal metabolome, and liver transcriptome data from the same individuals; thus, direct evidence linking specific microbial alterations to changes in butyrate or PAGln levels and subsequent host target regulation remains unavailable.

Future studies should prioritize experimental validation of the most strongly supported candidate interactions. At the clinical level, simultaneous collection of fecal samples, serum, and liver tissues from HBV- and NAFLD-associated fibrosis cohorts, combined with metagenomics, targeted metabolomics, and transcriptomics, would help clarify relationships among microbial functional pathways, butyrate/PAGln levels, and expression of HDAC3, ADRA2A, and chemokines. At the cellular level, primary hepatic stellate cells and hepatocyte–macrophage–stellate cell co-culture systems could be used with HDAC3 or ADRA2A perturbation, receptor antagonism, and metabolite supplementation to assess changes in α-SMA, COL1A1, inflammatory mediators, and chromatin acetylation. At the animal level, antibiotic depletion, germ-free models, microbiota transplantation, or defined microbial colonization approaches may further determine whether microbiota alterations influence fibrosis through specific metabolites. Establishing reproducible intervention and rescue relationships among metabolite changes, target engagement, signaling responses, and fibrotic phenotypes will be essential to transform the current candidate network into defined causal pathways.

## Conclusion

By integrating cross-etiology transcriptomics, protein–protein interaction networks, functional enrichment analysis, molecular docking, molecular dynamics simulations, and Mendelian randomization, this study identified 14 gut microbiota-associated core genes involved in liver fibrosis. These genes were primarily linked to immune chemotaxis, IL-17/TNF inflammatory signaling, epigenetic regulation, oxidative stress, and hepatic metabolic processes. Structural and genetic evidence highlighted ADRA2A–phenylacetylglutamine and butyrate–HDAC2/HDAC3 as potential microbiota metabolite–host target interactions, with HDAC3 emerging as a candidate node connecting microbial metabolic signals with liver fibrosis susceptibility. Our findings suggest that gut microbiota may regulate liver fibrosis through coordinated actions involving multiple metabolites, targets, and signaling pathways rather than a single linear axis. Further clinical multi-omics studies and experimental validation are required to establish these candidate mechanisms as causal pathways.

## Data Availability

The original contributions presented in the study are included in the article/**Supplementary Material**. Further inquiries can be directed to the corresponding authors.

## Supplementary material

Supplementary Figure 1 Molecular docking analysis of butyrate with HDAC2 and HDAC3. HDAC2/3, histone deacetylase 2/3. Docking results indicate potential structural compatibility between butyrate and HDAC proteins and do not represent experimentally confirmed direct binding. **(A)** Overall docking conformation of butyrate with HDAC2 (PDB ID: 4LXZ). The protein is shown as a blue cartoon representation and the ligand as an orange stick model. The predicted binding energy was −4.5 kcal/mol. **(B)** Three-dimensional interaction view of butyrate within the catalytic pocket of HDAC2, showing the spatial arrangement of the ligand and surrounding amino acid residues involved in predicted non-covalent interactions. **(C)** Two-dimensional interaction diagram of the butyrate–HDAC2 complex, showing hydrogen bonds, hydrophobic interactions, van der Waals contacts, and other predicted non-covalent interactions. **(D)** Overall docking conformation of butyrate with HDAC3 (PDB ID: 4A69). The protein is shown as a blue cartoon representation and the ligand as an orange stick model. The predicted binding energy was −4.4 kcal/mol. **(E)** Three-dimensional interaction view of butyrate within the catalytic pocket of HDAC3, showing the spatial arrangement of the ligand and surrounding amino acid residues involved in predicted non-covalent interactions. **(F)** Two-dimensional interaction diagram of the butyrate–HDAC3 complex, showing hydrogen bonds, hydrophobic interactions, van der Waals contacts, and other predicted non-covalent interactions. Dashed lines indicate predicted interactions.
